# Recognizing the Continuous Nature of Expression Heterogeneity and Clinical Outcomes in Clear Cell Renal Cell Carcinoma

**DOI:** 10.1038/s41598-017-07191-y

**Published:** 2017-08-04

**Authors:** Xiaona Wei, Yukti Choudhury, Weng Khong Lim, John Anema, Richard J. Kahnoski, Brian Lane, John Ludlow, Masayuki Takahashi, Hiro-omi Kanayama, Arie Belldegrun, Hyung L. Kim, Craig Rogers, David Nicol, Bin Tean Teh, Min-Han Tan

**Affiliations:** 1 Institute of Bioengineering and Nanotechnology, 31 Biopolis Way, The Nanos, 138669 Singapore Republic of Singapore; 2 Lucence Diagnostics Pte Ltd, Singapore, Republic of Singapore; 3 Cancer Stem Cell Biology Program, Duke-NUS Graduate Medical School, 8 College Road, Singapore, 169857 Republic of Singapore; 4 Urologic Consultants, 25 Michigan Street, Suite 3300, Grand Rapids, MI 49503 USA; 5 Division of Urology, Spectrum Health Medical Group, 4069 Lake Drive SE, Suite 313, Grand Rapids, MI 49546 USA; 6 Western Michigan Urological Associates, 577 Michigan Avenue, Suite 201, Holland, MI 49423 USA; 7 Department of Urology, Institute of Biomedical Sciences, Tokushima University Graduate School, 3-18-15, Kuramoto-cho, Tokushima, 770-8503 Japan; 8 FACS, Institute of Urologic Oncology, Department of Urology, David Geffen School of Medicine, University of California Los Angeles, 66-118 Center for Health Sciences Box 951738, Los Angeles, CA 90095 USA; 9 Division of Urology, Cedars-Sinai Medical Center, 8635W. Third Street, Suite 1070, Los Angeles, CA 90048 USA; 10 Vattikuti Urology Institute, Henry Ford Hospital, 2799W. Grand Blvd, Detroit, MI USA; 11 Department of Urology, The Royal Marsden NHS Foundation Trust, 203 Fulham Road, London, SW3 6JJ UK; 12 The Institute of Cancer Research, 123 Old Brompton Road, London, SW7 3RP UK; 13 Laboratory of Cancer Epigenome, National Cancer Centre Singapore, 11 Hospital Drive, Singapore, 169610 Republic of Singapore; 14 Cancer Science Institute of Singapore, National University of Singapore, 14 Medical Drive, #12-01, Singapore, 117599 Republic of Singapore; 15 Division of Medical Oncology, National Cancer Centre Singapore, 11 Hospital Drive, Singapore, 169610 Republic of Singapore; 16 MRL IT, MSD International GmbH (Singapore Branch), 1 Fusionopolis Place, #06-10/07-18, Galaxis, Singapore, 138522 Republic of Singapore

## Abstract

Clear cell renal cell carcinoma (ccRCC) has been previously classified into putative discrete prognostic subtypes by gene expression profiling. To investigate the robustness of these proposed subtype classifications, we evaluated 12 public datasets, together with a new dataset of 265 ccRCC gene expression profiles. Consensus clustering showed unstable subtype and principal component analysis (PCA) showed a continuous spectrum both within and between datasets. Considering the lack of discrete delineation and continuous spectrum observed, we developed a continuous quantitative prognosis score (Continuous Linear Enhanced Assessment of RCC, or CLEAR score). Prognostic performance was evaluated in independent cohorts from The Cancer Genome Atlas (TCGA) (n = 414) and EMBL-EBI (n = 53), CLEAR score demonstrated both superior prognostic estimates and inverse correlation with anti-angiogenic tyrosine-kinase inhibition in comparison to previously proposed discrete subtyping classifications. Inverse correlation with high-dose interleukin-2 outcomes was also observed for the CLEAR score. Multiple somatic mutations (VHL, PBRM1, SETD2, KDM5C, TP53, BAP1, PTEN, MTOR) were associated with the CLEAR score. Application of the CLEAR score to independent expression profiling of intratumoral ccRCC regions demonstrated that average intertumoral heterogeneity exceeded intratumoral expression heterogeneity. Wider investigation of cancer biology using continuous approaches may yield insights into tumor heterogeneity; single cell analysis may provide a key foundation for this approach.

## Introduction

Clear cell renal cell carcinoma (ccRCC) is a heterogeneous disease with diverse morphologies, molecular characteristics, clinical outcomes and therapeutic responses^1, 2^. Clinicopathologic prognostic factors including tumor stage, nuclear grade, morphologic characteristics, tumor size, nodal status and patient performance status^3–5^ have been studied as features to classify and predict disease-specific survival. However, these prognostic factors have limited accuracy for tumors of intermediate grade and stage that form a substantial proportion of ccRCC cases.

To improve prognostic prediction, several studies have proposed dividing ccRCC into molecular subtypes, primarily through unsupervised clustering of gene expression profiles or genetic alterations. Although these methods were reported to yield good performance in predicting survival, the results may be susceptible to instability arising from the analytic techniques used^6, 7^. There is also insufficient rigorous testing and evaluation of the performance of these subtype classification approaches in independent datasets^8–15^.

In this study, we examined 12 ccRCC datasets using consensus clustering sensitivity analysis by varying key parameters including item, feature, distance and iteration. We found that the clustering techniques yielded relatively unstable sample classification. Based on the observed lack of clear delineation between prognostic subtypes, we recognized that a genetic continuum would possibly reflect underlying biologic, histopathologic and clinical heterogeneity better than subtyping. To reflect this probable underlying reality, we developed an alternative strategy involving a continuous quantitative expression assessment of ccRCC tumor prognosis using tumor grade, which is a key histopathologic parameter in determining tumor behavior across different cancers. We thus created a continuous CLEAR score (continuous linear enhanced assessment of ccRCC) based on 18-transcript signature derived from a large internal dataset. In applying it to multiple external datasets, we investigated the performance of the CLEAR score, as a continuous measure of intertumoral expression heterogeneity, in estimating survival outcomes as compared to subtyping approaches. We evaluated outcomes of anti-angiogenic tyrosine-kinase inhibition, as well as high-dose interleukin-2 (IL-2) therapy in relation to this continuous CLEAR score. We further applied the CLEAR score to investigate 65 heterogeneous tumor regions from 10 ccRCC patients to determine extent and importance of intratumoral expression heterogeneity. The CLEAR score package is available at: https://sourceforge.net/user/verification?hash = 3dfcf804138b6b959a315335e0203a19.

## Results

### Evaluation of ccRCC subtyping with public datasets

To investigate the performance of ccRCC subtyping, 12 public datasets were obtained from the GEO database, EMBL-EBI and The Cancer Genome Atlas (TCGA) (Supplementary Table S1). Sensitivity analysis of consensus clustering done through varying key parameters demonstrated a discrepant number of predicted clusters ranging from 2 to 4 (Supplementary Table S2). Analysis by principal component analysis (PCA) showed a continuous spectrum (Supplementary Figures S1, S2).

### Assessment of 265 internal ccRCC samples with the CLEAR score

**Figure 1:**
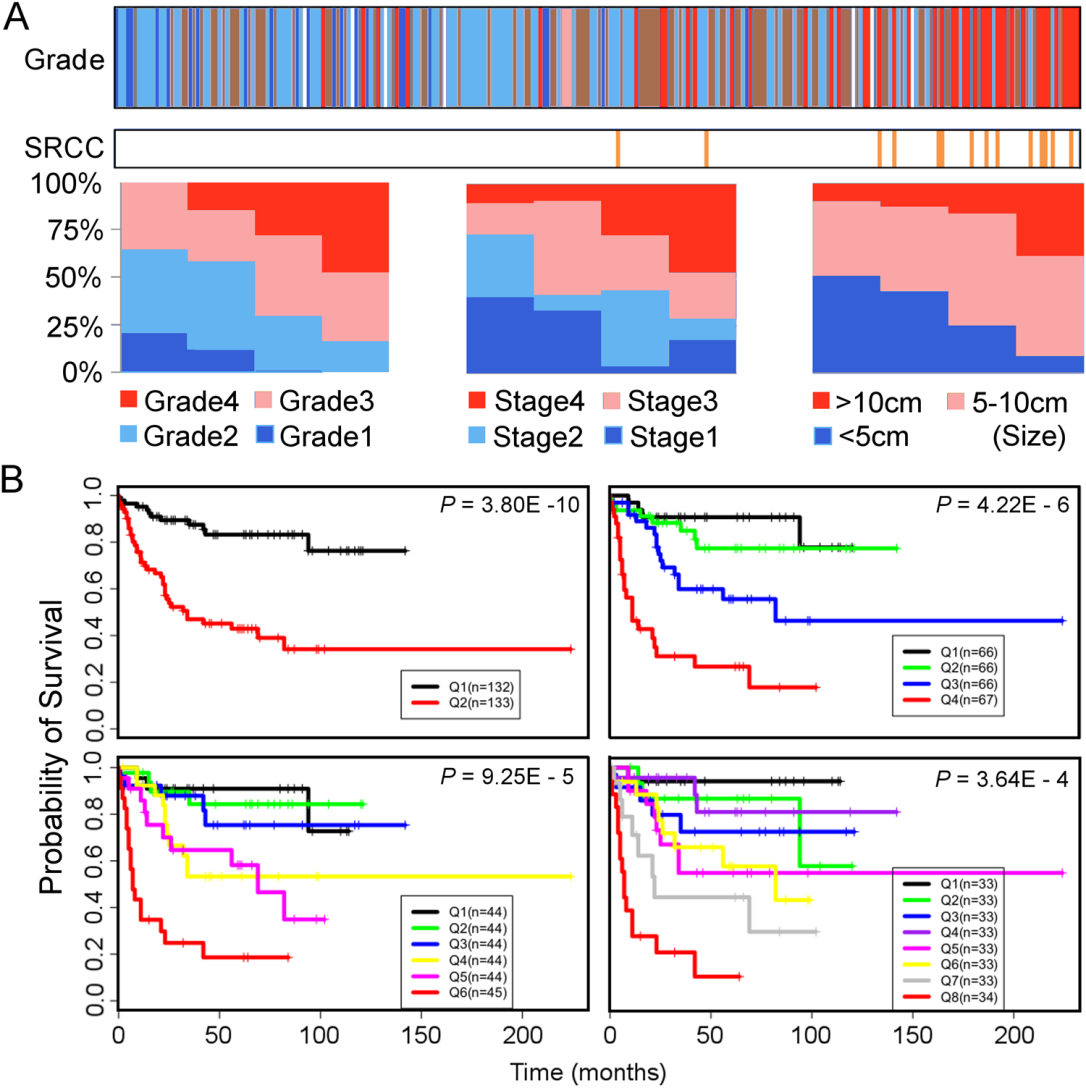
Validation of continuous linear enhanced assessment of ccRCC (CLEAR) by correlation and survival analysis. (**A**) Validation of CLEAR approach by correlation analysis with clinical variables. 265 ccRCC samples are ranked in ascending order based on CLEAR score. The top panel color bar represents the grade distribution profile of 265 ccRCC samples, expressed as colored vertical bars (white bars represent absent data). An association between high grade tumors and high CLEAR scores is observed (Fisher’s exact, p = 7.594e-07). A similar association between sarcomatoid RCC (SRCC) and high CLEAR scores is observed in the second horizontal bar (Mann-Whitney U test, p = 1.38e-06). To depict association with other key clinical variables, samples were evenly separated into 4 subgroups along the ranking queue with bar charts showing proportional breakdown of clinical variables. Correlation of tumor stage, tumor size with CLEAR score were observed (Fisher’s exact, p = 5.892e-05; Mann-Whitney U test, p = 1.10e-05, respectively). (**B**) Kaplan-Meier curves of cancer-specific survival for 265 ccRCC samples. We evenly grouped 265 ccRCC samples along the CLEAR scale into two (Q1 and Q2 groups), four (Q1–Q4 groups), six (Q1–Q6 groups) and eight (Q1–Q8 groups). In addition to inspection, Kaplan-Meier analysis was used to evaluate the association of these subgroups with survival, demonstrating a significant correlation with cancer-specific survival at all subgroupings.

In light of the above results, we hypothesized that a continuous scale might be more representative of tumor biology. The CLEAR score algorithm (Supplementary Figure S3) was thus designed. With our internal 265 ccRCC gene expression profiles, the continuous scale were derived by calculating the correlation distance between the sample of interest and two reference sample sets (RSSs) with distinct tumor grade (RSS1 with tumor grade 1 sample sets and RSS2 with tumor grade 4 sample sets). This scale was then normalized (range from 1 to 100) and defined as CLEAR score (Supplementary Dataset S1). We determined that various clinical variables correlating with biological aggressiveness such as grade, stage and size distributed continuously across the CLEAR score (Fig. 1A). Sarcomatoid histology of ccRCC is correlated with higher CLEAR score, with 9 of 14 samples having a score exceeding 80 (Supplementary Table S3). Corresponding survival analysis of these 265 ccRCC yielded varying cancer-specific survival outcomes across the CLEAR score (p < 1e-02) (Fig. 1B).

### Derivation of molecular drivers of CLEAR score and validation in TCGA datasets

**Figure 2:**
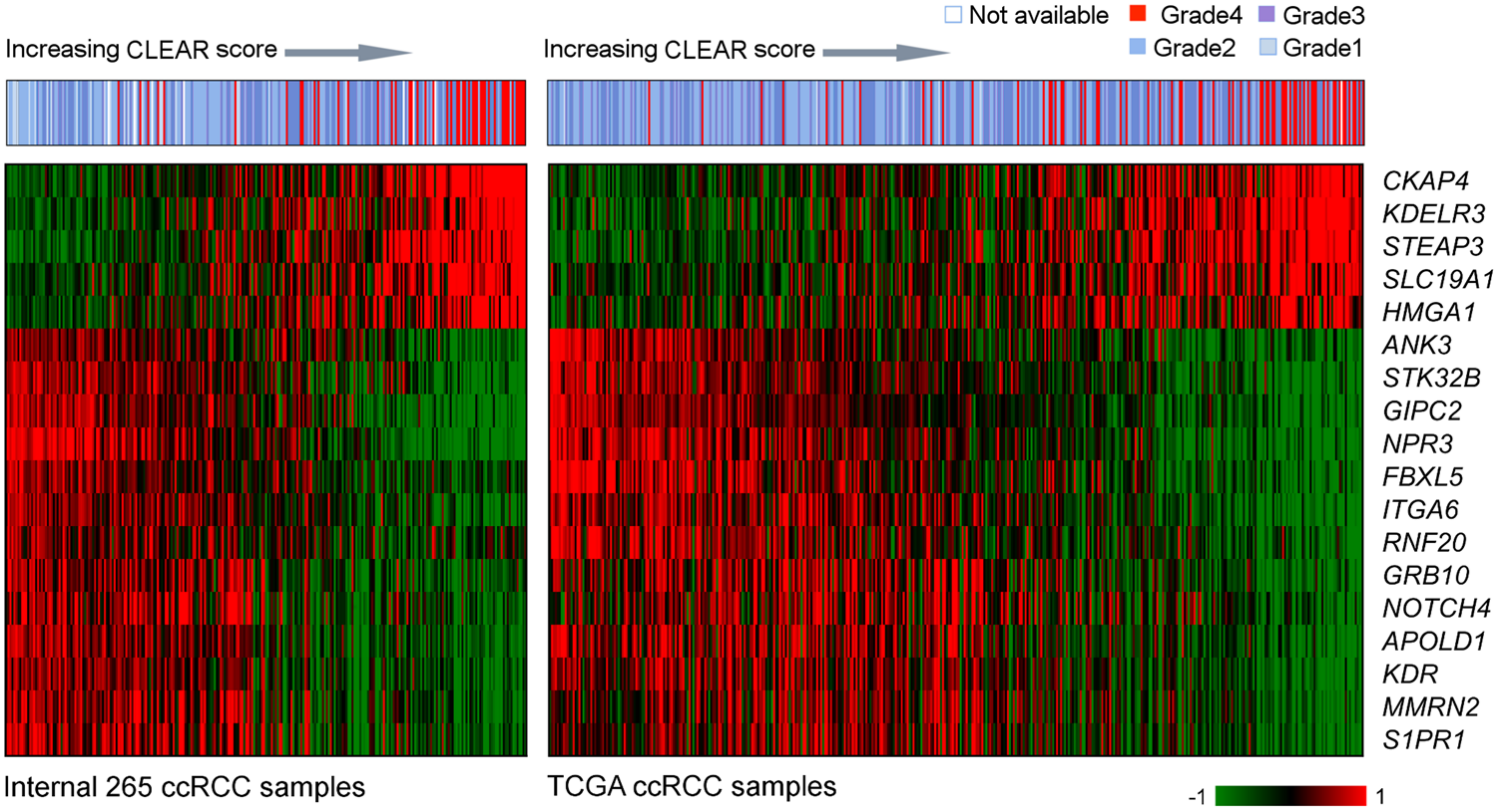
Heatmaps of 18-transcript signature profiles from internal TCGA cohort. The top panel color bar represents the tumor grade distribution profile as determined by the 18-transcript signature. Heatmaps below the color bar are gene expression profiles from cohorts of internal 265 samples and 414 TCGA ccRCC samples. Samples are ranked in ascending order based on CLEAR score. We observe that signature genes show a consistent expression pattern in these two cohorts. A similar association between CLEAR score and tumor grade was observed.

**Figure 3:**
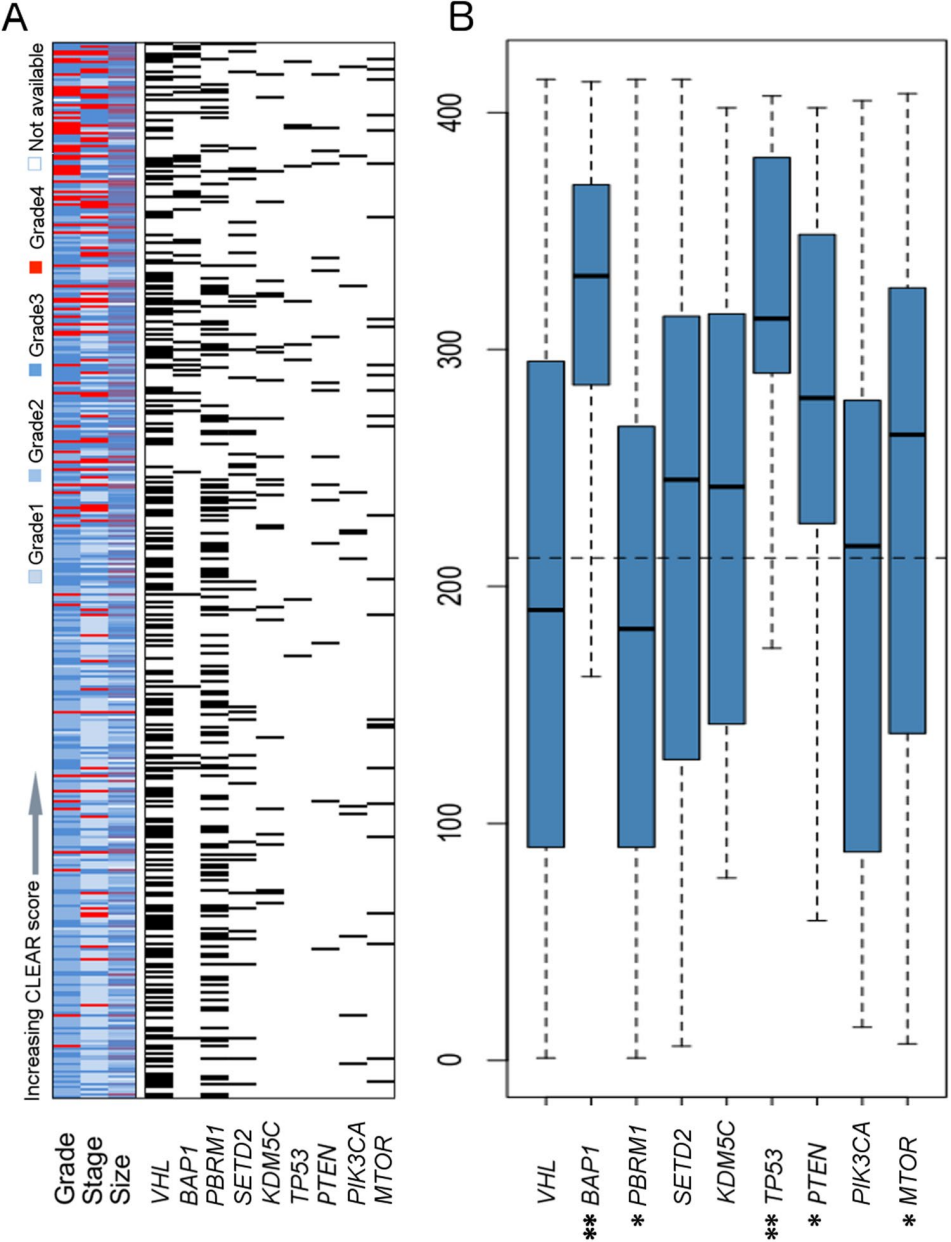
Correlation of CLEAR score and mutation in TCGA cohort. (**A**) Individual correlation between tumor CLEAR score with tumor grade, stage and size distribution as well as mutation status are presented. The correlation of CLEAR score and TCGA grade, stage was investigated by ANOVA (p = 7.769e-09, p = 3.22e-08, respectively); the correlation of CLEAR score and TCGA sample size was estimated by spearman correlation (p = 4.252e-09). (**B**) Boxplots of CLEAR score distribution of samples with relevant gene mutations. The dashed line represents the median CLEAR score of the 414-sample set. Samples with mutated BAP1, TP53, PTEN and MTOR are associated with higher CLEAR scores (Mann–Whitney U test, *p ≤ 0.05, **p ≤ 0.001”).

A signature derivation method based on correlation of CLEAR score with gene expression was developed (Supplementary Figure S4), yielding an 18-transcript signature. To independently evaluate the performance of the CLEAR score, we applied it to the TCGA-414 cohort^15^ generated by the RNA-seq. The expression profiles of 18-transcript signature exhibited a similar expression pattern between our internal dataset and independent TCGA dataset (p = 8.5e -07, spearman correlation) as presented in Fig. 2. An association between tumor advanced grade, stage and size with high CLEAR score were observed in Fig. 3A with TCGA dataset. The correlation of somatic events with CLEAR score using TCGA dataset was further investigated in the following section (Fig. 3B). Kaplan-Meier survival analysis presented a significant difference in cancer-specific survival among gradient groups (p < 1e-02) (Supplementary Figure S5). We further evaluated 11 additional public ccRCC datasets using the same approach and CLEAR score for each sample are available upon request.

### Comparison of CLEAR with the prognostic subtype model

**Figure 4:**
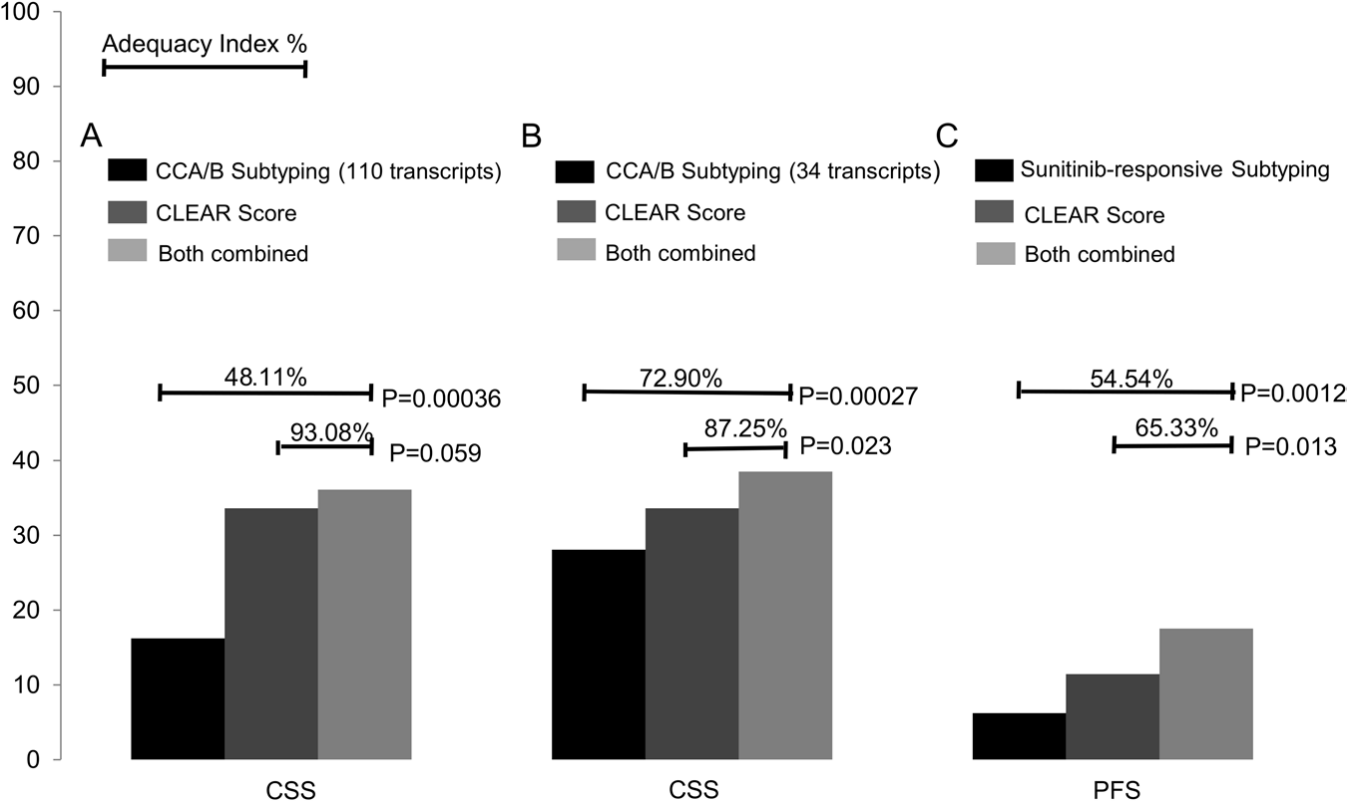
Predictive values for CLEAR Score model versus prior models. The predictive value is represented by the likelihood ratio test and adequacy index. The adequacy index is a measure of the evaluated model (as a subset of the full model), represented as a % relative to the full model, which does include both subtype and CLEAR Score model). (**A**) Predictive value of CCA/B subtyping (110 transcripts) (Gulati *et al.)* and CLEAR score model for cancer-specific survival (CSS) using TCGA dataset. (**B**) Predictive value of CCA/B subtyping (34 transcripts) (Brooks *et al*.) and CLEAR score model for CSS using TCGA dataset. (**C**) Predictive value of sunitinib-responsive subtyping (Beuselinck *et al*.) and CLEAR Score model for progression free survival (PFS) using E-MTAB-3267 datasets. These three comparison result showed the addition of the CLEAR score model to one containing the CCA/B subtype model significantly improves the predictive value of the final model, but there is no significant difference when the subtype model is added to the CLEAR model.

Two sets of gene expression signatures (110 transcripts and 34 transcripts respectively) have been previously identified for prognostic classification^13, 16, 17^. To evaluate the prognostic accuracy of CLEAR score for survival outcomes, we compared the predictive value of CLEAR score model with these two prognostic models of CCA/CCB classification^16, 17^ using the independent TCGA dataset. Likelihood ratio testing^18^ demonstrated a significant benefit in prediction when adding the CLEAR score model to these two CCA/B subtyping models (110 transcripts and 34 transcripts respectively) in cancer-specific survival tested, with no significant benefit using the converse approach (CSS: p = 0.00036 vs. p = 0.059; CSS: p = 0.00027 vs. p = 0.023). The higher adequacy index of the CLEAR model demonstrated that the expression-based continuous CLEAR score provide improved performance (Fig. 4A,B).

### Association of CLEAR score with therapeutic response

To investigate the potential utility of the CLEAR approach in predicting patient benefit from TKI treatment, we applied CLEAR to a public dataset (E-MTAB-3267) with 53 metastatic French ccRCC patients who received first-line sunitinib^19, 20^. Patients who experienced a complete or partial response (CR/PR) presented a relatively lower CLEAR score than patients with progressive disease (PD), who presented with a very high median score (Mann–Whitney U test, p = 0.000149) (Supplementary Figure S6). We further compared the predictive value of CLEAR with the model reporting association of subtypes with sunitinib response. Likelihood ratio testing demonstrated a superior performance of the CLEAR score to the proposed molecular subtyping in predicting outcomes (PFS: p = 0.0012 vs p = 0.013) (Fig. 4C). We further evaluate correlation of CLEAR score with response to sunitinib by wald test and logrank test in coxph model (p = 0.0006958, p = 0.0004619, respectively). Similarly, CLEAR score with response to IL-2 were also evaluated with coxph model by wald test and logrank test (p = 0.00032, p = 0.00076, respectively).

It is notable that several patients in the internal dataset received high-dose IL-2 treatment, of which 4 and 6 patients experienced durable complete response and eventual progressive disease respectively. High dose IL-2 is associated with durable responses in metastatic ccRCC^21^ but is associated with significant toxicities. In general, all patients undergoing high-dose IL-2 therapy had relatively high CLEAR scores (above 50). While sample numbers were low, there was a trend to lower CLEAR scores in patients experiencing complete responses (Mann–Whitney U test, p = 0.05) (Supplementary Table S4, Supplementary Figure S7).

### Assessment of Intratumoral expression heterogeneity

**Figure 5:**
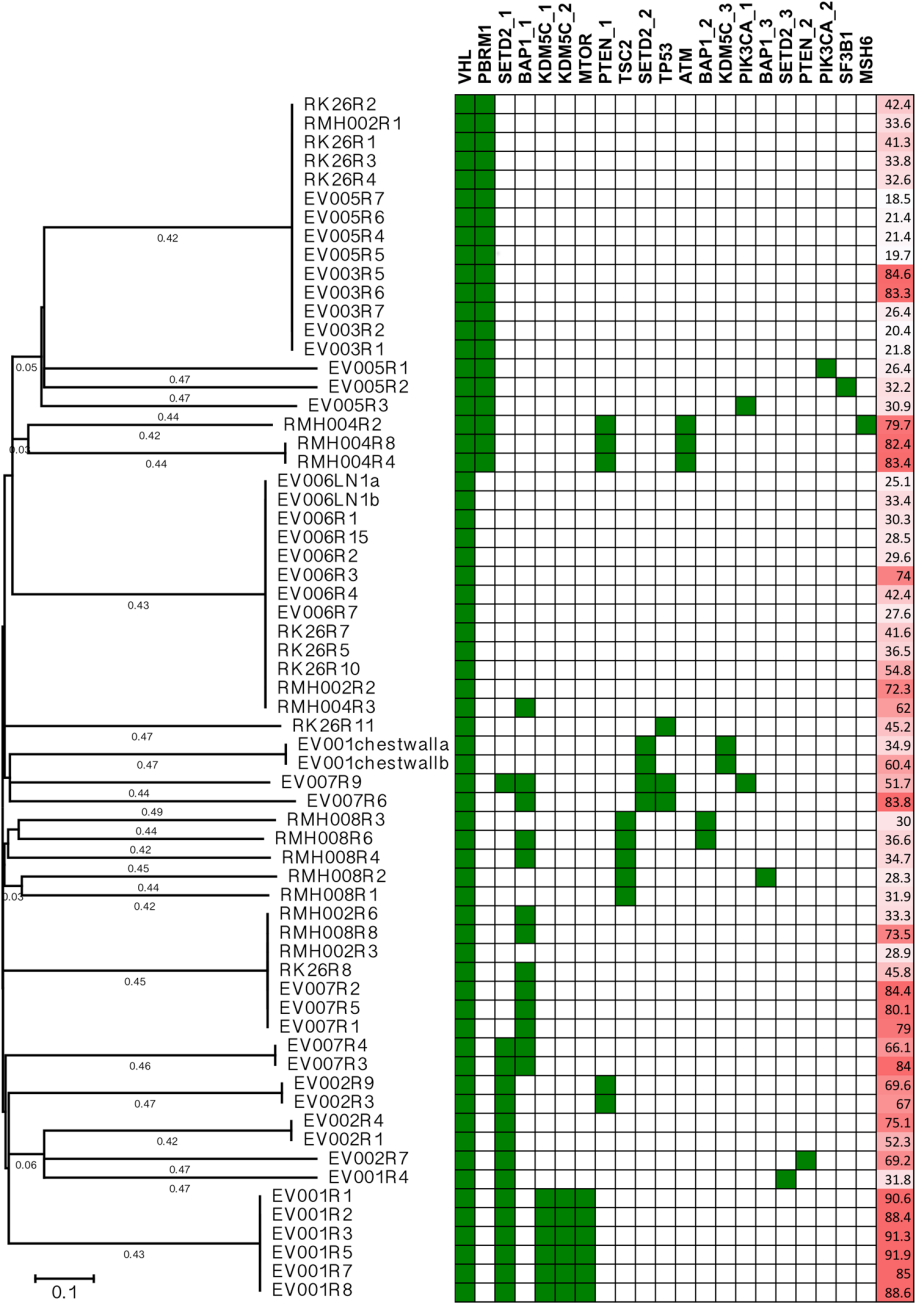
Phylogenetic tree with driver mutation status and CLEAR score. The region tree provides the information of the connection of sample diversity, prognosis and the driver gene mutation. The region tree shows majority of sample regions are clustered together with their siblings with several exceptions caused by intra-tumor heterogeneity and PBRM1 is associated with regions with low prognosis score, while SETD2, BAP1 and KDM5C are associated with the poor prognosis sample regions.

The CLEAR score can be applied on different regions of the same tumor. We investigated intratumoral expression heterogeneity using the GSE53000 dataset from GEO database, which contained 65 regions from 10 ccRCC patients^22, 23^. Supplementary Dataset S2 presents the CLEAR score for all 65 regions. The boxplot in Supplementary Figure S8 shows the distribution of CLEAR score which indicated that when the median CLEAR score increase, the subsamples tend to have CLEAR score that are more divergent (one sample exception of EV003). The correlation of median absolute deviation (MAD) with CLEAR score from 10 ccRCC patients were investigated by Pearson correlation (P = 0.02886), thus then MAD of CLEAR score in each patient was used as an indicator for measurement of the variation of heterogeneity (Supplementary Table S5). To examine intertumoral and intratumoral expression heterogeneity, gene expression profiles of the with 18-transcript signature from 65 regions of 10 individual tumors^22, 23^ and 414-TCGA samples were summarized into a heatmap and all the samples ordered based on CLEAR score (Supplementary Figure S9). The MAD of CLEAR score of all these 65 regions is much larger than the regions within individual tumors, suggesting that intertumoral expression heterogeneity commonly exceeds intratumoral expression heterogeneity (ANOVA, p = 6.29e-03). EV005, RMH008, EV006, EV003 have the lower degree of intratumoral expression heterogeneity while EV001, EV007, RK26, RMH002, EV003, RMH004 has the higher degree of intratumoral expression heterogeneity (Fig. 5, Supplementary Table S5).

### Correlation of CLEAR score with somatic mutations

To investigate the correlation of CLEAR score with intertumoral mutational heterogeneity, after identifying the CLEAR score of TCGA cohort, we examined the reported ccRCC somatic mutation events per sample (Fig. 3A) and the strong association of ccRCC mutation with CLEAR score was observed (ANOVA test, p = 1.18E-08). Further analysis showed *BAP1, TP53*, *PTEN* and *MTOR* were associated with higher CLEAR scores (Mann–Whitney U test, *BAP1* (p = 2.32e -08); *TP53* (p = 0.0004); *PTEN* (p = 0.009); *MTOR* (p = 0.04)). In contrast, an association between lower CLEAR scores and somatic mutations of *PBRM1* (p = 0.045) were noted (Fig. 3B).

We further evaluated the correlation of CLEAR score with mutation in intratumoral mutational heterogeneity using the GSE53000 dataset^22, 23^. A sample region tree with the driver mutation information was constructed using the maximum parsimony method (Fig. 5). The phylogenetic tree was then annotated with driver gene mutation status and CLEAR score. From the region tree, we can observe most sample regions from the same tumor clustered together with several exceptions caused by intratumoral expression heterogeneity, which indicates that the degree of intertumoral heterogeneity is larger than that of intratumoral expression heterogeneity. Our result also showed that *PBRM1* is associated with regions having low CLEAR score, while *SETD2*, *BAP1* and *KDM5C* are associated with the sample regions which have higher CLEAR score.

## Discussion

In our evaluation of 12 independent datasets, molecular subtyping as derived by unsupervised methods yielded varying subtypes depending on parameters. To reflect the apparent continuum as observed in data analysis, the CLEAR score was designed as a continuous quantitative score derived from histopathologic tumor grade, and corresponding to tumor aggressiveness of ccRCC. Its application to samples allows for evaluation of intertumoral heterogeneity on a continuous scale. Investigation of its performance in multiple independent datasets shows better predictions of prognosis and drug response in comparison to proposed subtyping, suggesting that intertumoral heterogeneity as measured by CLEAR scoring on a continuum is biologically and clinically meaningful. It is of considerable interest that the CLEAR score here, founded on a relatively simple pathology-based morphologic approach, outperformed complex supervised and unsupervised analyses, implying that careful consideration of appropriate pathologic data for integrated analyses may be of meaingful benefit and insights. We speculate that a broader application of this approach to other cancer types may yield helpful insights. The CLEAR score may either be interpreted as a varying mixture of a composite of cells on two extremes of biological aggressiveness, or a clonal expansion of cells that vary across expression heterogeneity. While evaluation of matching CLEAR scores in different regions of the same tumor may help to address such questions, single-cell expression analysis would allow a definitive conclusion.

Gene expression and deep sequencing analysis have provided insights into clinical heterogeneity^22, 23^. Corresponding reports have suggested heterogeneous expression patterns with good and poor prognosis signatures coexisting in 8 of 10 cases^16^. Research into intratumoral expression heterogeneity and its corresponding real-world clinical implications such as drug resistance, are expanding rapidly. The use of the CLEAR score can provide a strong basis for quantifying such variation, thus supporting development of improved diagnostics and therapeutics. Our work has provided direct empirical support for an observation that average intertumoral bulk expression heterogeneity exceeds intra-tumoral heterogeneity, which has not been previously demonstrated. Additional research investigating the origin of such apparent heterogeneity in clonal dynamics or deriving from an ancestral ccRCC clone will have profound implications for drug development. Indeed, we speculate the observed association between improved drug response and lower CLEAR score may reflect improved outcomes in tumors with lower heterogeneity.

The distinction between a prognostic and predictive biomarker of treatment is important. Beyond a correlation with prognostic outcomes in patients with localized disease, the CLEAR score exhibits an inverse correlation with outcomes of high-dose IL-2 treatment and anti-angiogenic tyrosine kinase inhibition (TKI) in patients with metastatic RCC. This indicates that the CLEAR score is likely to be a predictive biomarker, the nature of which is best tested in a prospective clinical trial. Given the anti-angiogenic and immune-related mechanisms of each drug class, research to dissect the immune and stromal contributions to tumor gene expression may shed more light on this association.

The limitations of this study are its retrospective design that might influence sample selection, and the relatively limited number of subjects for IL-2 and TKI response association analysis. However, given the strong external validation in a wide range of data-sets and platforms, also including patients undergoing TKI treatment, we believe that our results are generalizable. Additional validity may be observed by its excellent performance when applied across platforms on RNA-Sequencing data, which would be of interest in future translation.

In summary, we report that a continuous quantitative scoring in ccRCC derived through conventional pathologic parameters yielded improved biological and clinical predictions over dichotomous subtype classifications, suggesting expression heterogeneity and corresponding biology is more suitably determined on a continuous scale. When applied to intratumoral regions, the CLEAR score suggests intertumoral heterogeneity generally exceeds intratumoral expression heterogeneity.

## Materials and Methods

### Public data collection

To evaluate ccRCC subtypes, public datasets were collected from GEO, EMBL-EBI and TCGA (The Cancer Genome Atlas database) with the key words renal cell carcinoma or ccRCC. A total of 12 ccRCC microarray and RNA-seq data were collected (Supplementary Table S1). Consensus clustering sensitivity analysis was used to evaluate ccRCC subtypes from these 12 ccRCC datasets. Principal component analysis (PCA) and clustering typing with the well selection of reported ccRCC markers^13^ were further applied to examine the appearance of ccRCC subtypes.

### Sample processing and data analysis

Expression profiles of 265 ccRCC samples were obtained from the Van Andel Research Institute. Baseline characteristics of patients are described in Supplementary Table S6. Gene expression profiles of these 265 samples were obtained using the HG-U133_Plus_2 platform. The resultant expression data was then summarized and normalized using the Robust Multi-array Average (RMA) method of the R “simpleaffy” Package (http://www.r-project.org). The expression data of 265 ccRCC samples were deposited in the Gene Expression Omnibus (GEO) under the accession number of GSE73731 (http://www.ncbi.nlm.nih.gov/geo/query/acc.cgi?token=udstaaakhbubjkt&acc=GSE73731).

### CLEAR Algorithm

We first applied an unbiased filtering method to keep genes with log expression values greater than 8 in at least 10% of the samples, allowing for 792 genes used in model training. The analytic pipeline of CLEAR is illustrated in Supplementary Figure S3. The CLEAR approach uses two reference sample sets (RSSs) defined by clinical parameters as two extremes. The RSS sets are defined as sample sets that represent two distinct states that relate to a clinical factor, for example, tumor grade 1 sample sets and tumor grade 4 sample sets. Tumor grade is a key morphologic and pathologic correlate of tumor biology. We marked these two RSSs as RSS1 (tumor grade 1 sample set) and RSS2 (tumor grade 4 sample set) respectively. We then computed the average gene expression of these two RSSs to yield two median expression profiles **Expr** (RSS1) and **Expr** (RSS2) with a gene set **G** with *N* gene values (for our case, *N* = 7924). These two RSSs served as reference centroids to determine the relative scale location of a testing sample between these two centroids.

For testing an individual sample S_*k*_ with expression **Expr**, we sampled *m* subset of genes with the bootstrapping strategy: **M** = {**g**_**1**_ **, g**_**2**_ **, …, g**_**m**_}. Then, for each **g**_**i**_ **€ M**, we computed the correlation distance between Sk and RSS1 and RSS2 respectively as:

$${\rm{Dist}}({{\rm{S}}}_{k},{\rm{RSS}}1,i)={\rm{Corr}}({{\rm{S}}}_{k},{\rm{RSS}}1,{\bf{g}}i)$$

and

$${\rm{Dist}}({{\rm{S}}}_{k},{\rm{RSS}}2,i)={\rm{Corr}}({{\rm{S}}}_{k},{\rm{RSS}}2,{\bf{g}}i)$$

We then defined the CLEAR score^1^ as:

$${{\rm{CS}}}_{i}={{\rm{\Sigma }}}_{(1\le i\le m)}({\rm{Dist}}({{\rm{S}}}_{k},{\rm{RSS}}1,i)/{\rm{Dist}}({{\rm{S}}}_{k},{\rm{RSS}}2,i))/m,$$

which is the average correlation ratio computed from *m* bootstrapped gene sets **M**. For a sample set with *r* samples, the CLEAR score set will be **P** = {CS_1_, CS_2_, …, CS_*r*_}.

For a more intuitive view of the CLEAR score, we scaled score within the range [1,100] using the equation:

$${{\rm{S}}}_{i}=[({{\rm{CS}}}_{i}-\,\min ({\bf{P}}))/({\rm{\max }}({\bf{P}})-\,\min ({P)}^{\ast }99)]+1,$$

where **P** is the CLEAR score sets for all the samples. With the CLEAR score, we can determine to what extent the testing sample is similar to the two RSSs and then map this onto the hyperspace coordinate defined by the two RSSs that relate to a certain clinical factor.

To assess the variation of the CLEAR score with different RSSs, we selected all tumor grade 1 samples and tumor grade 4 samples. By using bootstrapping samples, 500 RSSs, each containing a unique combination of reference sample sets, were generated. Every RSS was then used to determine the CLEAR score based on our algorithm. For each sample, MAD (median absolute deviation) was used to evaluate the variation of the CLEAR score (Supplementary Figure S10).

### Validation of CLEAR score development by correlation and survival analysis

As described previously, we ranked the samples in ascending order based on their CLEAR score and defined this queue as the tumor ranking queue. To determine the correlation of the tumor ranking queue with clinical factors including tumor grade, stage and size, we separated 265 ccRCC samples into 4 subgroups evenly. In each subgroup, we calculated the percentage of samples with different levels of tumor grade, stage and size.

In an attempt to validate the algorithm using multiple clinical factors, we selected two other RSSs defined by distinct tumor stage and tumor size respectively. Spearman correlation coefficient by exact test was used to analyze the correlation between these three CLEAR scales. We further divided the 265 ccRCC samples along the CLEAR scale into 2, 4, 6 and 8 groups evenly. Kaplan-Meier analysis was used to evaluate the association of survival outcomes of these groups.

### Screening of gene signatures for CLEAR score

To screen potential genes that contribute to the CLEAR score and understand the underlying mechanism that drives the prognosis difference, a signature derivation method was developed in Supplementary Figure S5. As described in the last section, we ranked the samples in ascending order based on their CLEAR score determined with distinct tumor grade as RSSs and got the sample ranking queue 1. Similarly, sample ranking queue 2 and sample ranking queue 3 were determined with distinct tumor stage and tumor size as RSSs respectively. At the same time, for each gene, we created another ranking of samples according to the gene expression values. We then tested the correlation of these three sample ranking queues with the gene expression ranking using the Spearman and Kendall’s Tau correlation methods separately. The three most positively and negatively correlated gene sets were generated as potential candidate signatures and the top 50 common genes were selected. Using sensitivity analysis of the log rank test with cox proportional hazards modeling in relation to the CLEAR score, a signature was derived from the 18-signature transcript with the lowest p-values (Supplementary Figure S11).

### Comparison of CLEAR score and subtype model

To evaluate the predictive value of the CLEAR score model and the previously reported CCA/CCB subtyping model^13, 17, 19^ for a variety of survival outcomes, we used the likelihood ratio chi square test for nested models to assess whether the CLEAR score model adds predictive value to a model including the CCA/CCB. An adequacy index using likelihood ratio methods was used to quantify the percentage variation explained by a subset of the predictors (CLEAR or CCA/CCB separately) compared with the information contained in the full set of predictors (both CLEAR score and CCA/CCB) by means of log-likelihood^18, 24^. Pseudo R2 values derived from coxph was used to evaluate model performance (R Square of CCA/CCB model with 110 transcripts is 0.103; R Square of CCA/CCB model with 34 transcripts is 0.105; R Square of CLEAR model is 0.123). Similarly, the same method was used to test the predictive value of the CLEAR score model and Sunitinib-responsive subtyping with dataset of E-MTAB-3267^19^.

### Statistical analysis

We used the Fisher’s exact test for investigating the enrichment and depletion of advanced levels of tumor grade, stage and size in different subgroups along the CLEAR scale. Spearman and Kendall’s tau correlation tests were used for evaluation of candidate signatures that contributed to the tumor prognosis ranking. Spearman correlation coefficient by exact test was used to analyze the correlation of three ranking queue. Kaplan-Meier survival test was used to assess the association of cancer-specific survival with the sample subgroups. Significance was determined using the log rank test. Other clinical covariates including age, tumor stage and tumor grade were compared to the outcome using univariate and multivariate Cox proportional hazards modeling. Log rank and likelihood ratio tests were done for multivariate modeling to assess statistical significance. All tests were performed using the R statistical computing package (http://www.r-project.org).

## Electronic supplementary material

Supplementary Information

Dataset S1

Dataset S2

Dataset S3

Dataset S10
