# Supplementary material for: Recognizing the Continuous Nature of Expression Heterogeneity and Clinical Outcomes in Clear Cell Renal Cell Carcinoma
Source: Sci Rep. 2017 Aug 4;7:7342. doi: 10.1038/s41598-017-07191-y (PMC5544702; doi:10.1038/s41598-017-07191-y)
Supplement: Supplementary file 1 — Supplementary Information [file 41598_2017_7191_MOESM1_ESM.doc]

**Supplemental Information**

**Title:**  Recognizing the Continuous Nature of Expression Heterogeneity and Clinical Outcomes in Clear Cell Renal Cell Carcinoma

**Author:** Xiaona Wei1,2^, Yukti Choudhury^1^, Weng Khong Lim^3,14^, John Anema^4^, Richard J. Kahnoski^5^, Brian Lane^5^, John Ludlow^6^, Masayuki Takahashi^7^, Hiro-omi-Kanayama^7^, Arie Belldegrun^8^, Hyung L. Kim^9^, Craig Rogers^10^, David Nicol^11, 12^, Bin Tean Teh^3, 13, 14,*^, Min-Han Tan^1, 15,*^

**Author affiliation:**

^1^ Institute of Bioengineering and Nanotechnology, 31 Biopolis Way, The Nanos, Singapore 138669

^2^ Merck Research Labs IT, MSD International GmbH (Singapore Branch), 1 Fusionopolis Place, #06-10/07-18, Galaxis, Singapore 138522, Singapore

^3^ Cancer Stem Cell Biology Program, Duke-NUS Graduate Medical School, 8 College Road, Singapore 169857, Singapore

^4^ Urologic Consultants, 25 Michigan Street, Suite 3300, Grand Rapids, MI 49503

^5^ Division of Urology, Spectrum Health Medical Group, 4069 Lake Drive SE, Suite 313, Grand Rapids, MI 49546

^6^ Western Michigan Urological Associates, 577 Michigan Avenue, Suite 201, Holland, MI 49423

^7^ Department of Urology, Institute of Biomedical Sciences, Tokushima University Graduate School, 3-18-15, Kuramoto-cho, Tokushima, Japan 770-8503

^8^ FACS, Institute of Urologic Oncology, Department of Urology, David Geffen School of

Medicine, University of California Los Angeles, 66-118 Center for Health Sciences Box

951738, Los Angeles, CA 90095

^9^ Division of Urology, Cedars-Sinai Medical Center, 8635 W. Third Street, Suite 1070, Los Angeles, CA 90048

^10^ Vattikuti Urology Institute, Henry Ford Hospital, 2799 W. Grand Blvd., Detroit, MI

^11^ Department of Urology, The Royal Marsden NHS Foundation Trust, 203 Fulham Road, London SW3 6JJ, UK

^12^ The Institute of Cancer Research, 123 Old Brompton Road, London SW7 3RP, UK

^13^ Laboratory of Cancer Epigenome, National Cancer Centre Singapore, 11 Hospital Drive, Singapore 169610, Singapore

^14^ Cancer Science Institute of Singapore, National University of Singapore, 14 Medical Drive, #12-01, Singapore 117599, Singapore

^15^ Division of Medical Oncology, National Cancer Centre Singapore, 11 Hospital Drive, Singapore 169610

***** Corresponding Authors

^ Current Address

**Correspondence:**

Min-Han Tan, MBBS, FRCP, FAMS, PhD

Institute of Bioengineering and Nanotechnology, 31 Biopolis Way, The Nanos, #04-01
Singapore 138669

Fax number: +65 6478 9080; Telephone Number: +65 6824 7110

Email: [mhtan@ibn.a-star.edu.sg](mailto:mhtan@ibn.a-star.edu.sg)

**SI Guide**

Supplementary Figure S1: PCA analysis with public ccRCC datasets

Supplementary Figure S2: PCA analysis with public ccRCC datasets with top 20% variable gene subsets.

Supplementary Figure S3: Pipeline of CLEAR score algorithm

Supplementary Figure S4: Flowchart for signature derivation

Supplementary Figure S5: Kaplan-Meier curves of cancer-specific survival for 414 TCGA samples

Supplementary Figure S6: Boxplot of CLEAR score distribution in the patients with stable disease (SD), complete or partial response (CR/PR) and progressive disease (PD) with dataset of E-MTAB-3267

Supplementary Figure S7: Boxplot of CLEAR score distribution in the patients with complete response (CR) and progressive disease (PD) with IL-2 treatment in our dataset

Supplementary Figure S8: Assessment of CLEAR score of ITH of ccRCC

Supplementary Figure S9: Expression Heterogeneity and Clinical Outcomes in in ccRCC

Supplementary Figure S10: Variation of CLEAR score regarding to the different reference sample sets

Supplementary Figure S11: Sensitivity of Log-rank test with Cox proportional hazards model regarding to CLEAR Score

Supplementary Table S1: ccRCC datasets collected from GEO database, EMBL-EBI and TCGA

Supplementary Table S2: Summary of consensus clustering sensitivity analysis by varying

key parameters of repeat times, Pitem, Pfeature and distance

Supplementary Table S3: CLEAR score of Sarcomatoid Renal Cell Carcinoma samples.

Supplementary Table S4: CLEAR score of samples with IL-2 treatment

Supplementary Table S5: Median absolute deviation (MAD) of CLEAR score in patients using dataset of GSE53000

Supplementary Table S6: Clinical information of 265 datasets.

Supplementary Dataset S1: CLEAR score of GSE73731 (Internal dataset)

Supplementary Dataset S2: CLEAR score of GSE53000 (intratumoral samples).

Supplementary Dataset S3: CLEAR score of public datasets.


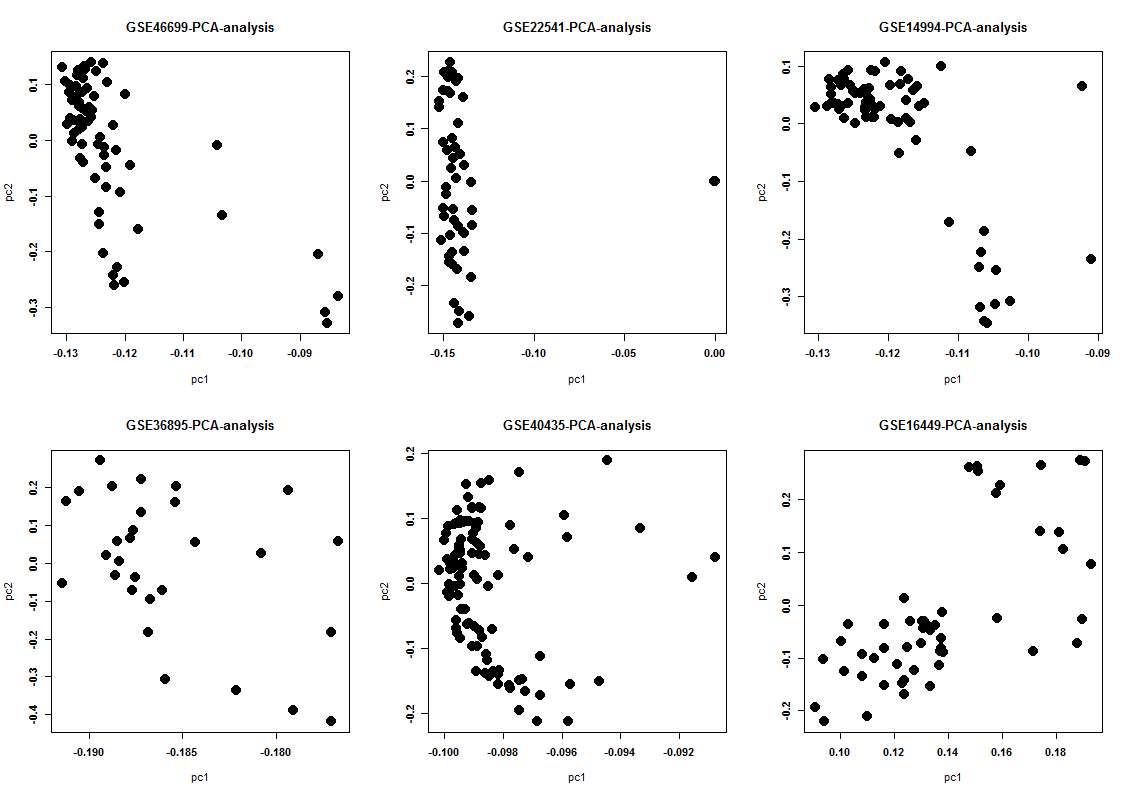


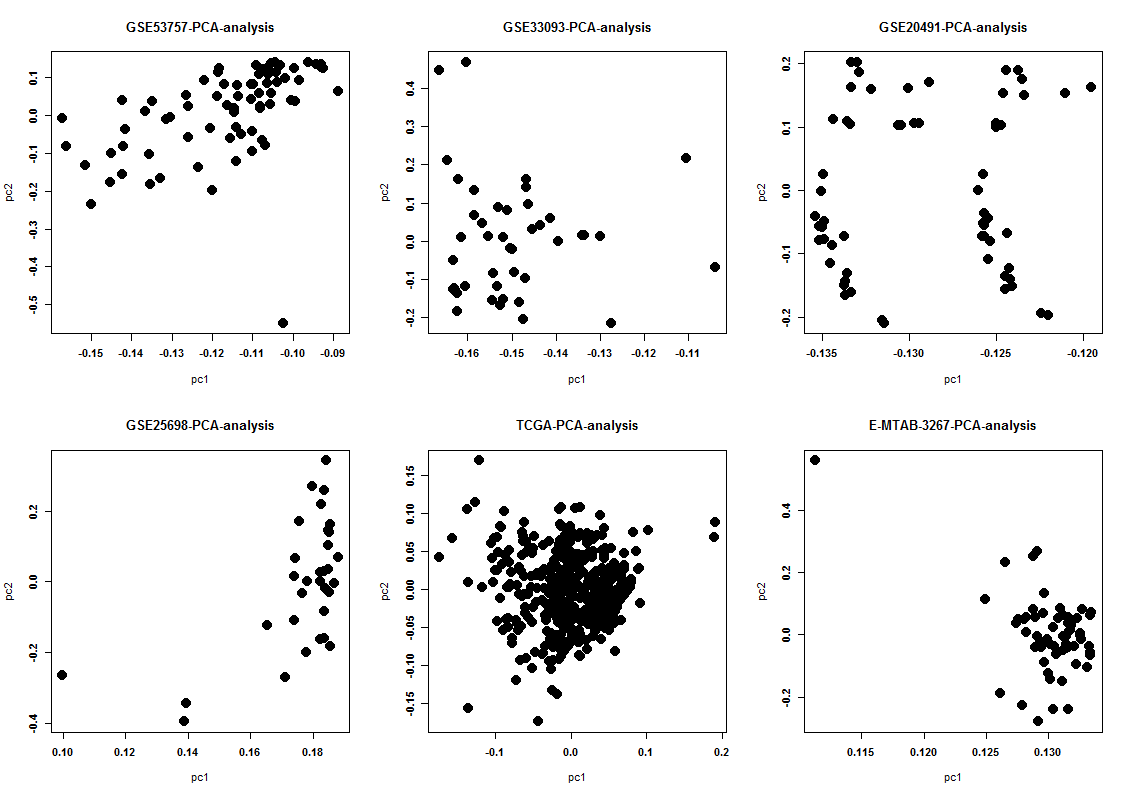

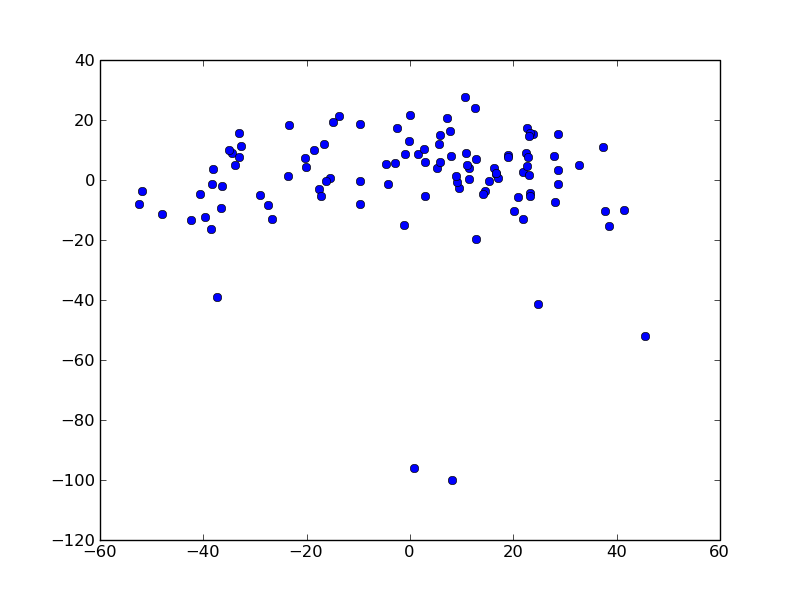

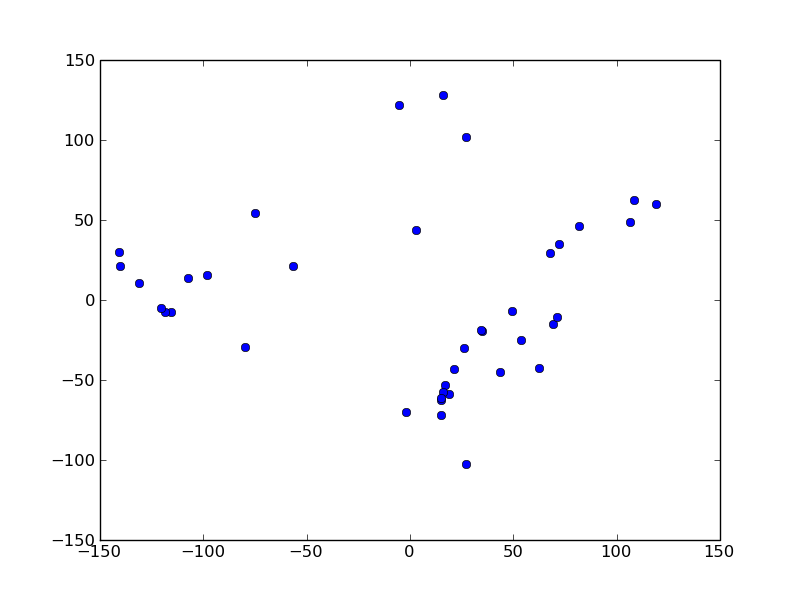


A

Supplementary Figure S1: PCA analysis with public ccRCC datasets with all probes or genes.


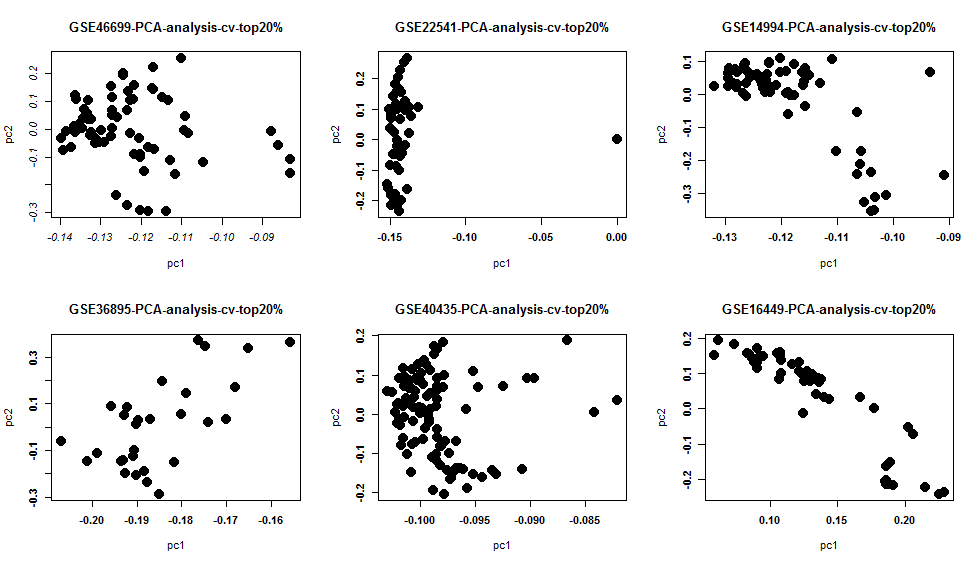


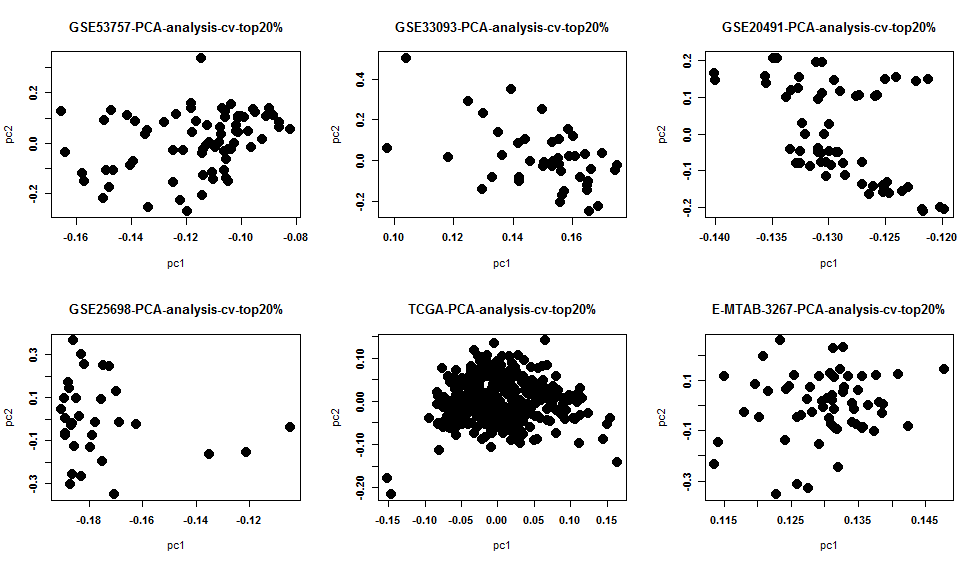


Supplementary Figure S2: PCA analysis with public ccRCC datasets with top 20% variable gene subsets.

Expression data

*M* Gene subsets

Bootstrapping of genes

*M* Median Expr. vectors of

RSS1 and RSS2

*M* Expr. vectors of sample *Sk*, (1≤*k*≤*N*)

*M* Expr. vectors of sample *Sk*, (1≤*k*≤*N*)

*M* Expr. vectors of sample *Sk*, (1≤*k*≤*N*)

Distance of S*k* to RSS1 and RSS2

*M* Expr. vectors of sample *Sk*, (1≤*k*≤*N*)

CS1= Dist(RSS1)/Dist(RSS2)

CS= (CS1+CS2……CS*m*)/*M*

Scale the score (Rang from 1 to 100)

Ranking the samples

*M* Expr. vectors of sample *Sk*, (1≤*k*≤*N*)

Supplementary Figure S3: Pipeline of CLEAR score algorithm.

* RSS: Reference Sample Sets with distinct clinical features

RSS 1 represent reference sample sets with distinct clinical features (tumor grade 1 or tumor stage 1). RSS 2 represent reference sample sets with distinct clinical features (tumor grade 4 or tumor stage 4).

S*k*: individual sample k. CS: Clear Score.

*M* Expr. vectors of sample *Sk*, (1≤*k*≤*N*)

Sample Scale 1 based on CLEAR Score

**R1**={r_1_, r_2_ ,..., r*_m_*}

Sample Scale based on CLEAR Score

**R2**={r_1_, r_2_ ,..., r*_m_*}

Sample Scale based on CLEAR Score

**R3**={r_1_, r_2_ ,..., r*_m_*}

Sample Rank based on gene expression

**R**’={r’_1_, r’_2_ ,..., r’*_m_*}

Sample Rank based on gene expression

**R**’={r’_1_, r’_2_ ,..., r’*_m_*}

Sample Rank based on gene expression

**R**’={r’_1_, r’_2_ ,..., r’*_m_*}

Correlation of **R**1and **R’**

by correlation test

Correlation of **R**2 and **R’**

by correlation test

Correlation of **R**3 and **R’**

by correlation test

Candidate signature sets1

Candidate signature sets2

Candidate signature sets3

Common top 50 Candidate signature sets

Sample Rank based on gene expression

**R**’={r’_1_, r’_2_ ,..., r’*_m_*}

Sensitivity analysis of log rank test

18 Signatures were derived

Supplementary Figure S4: Flowchart for signature derivation. R1, R2, R3 represented three CLEAR Scales which determined with three different reference sample sets, specifically, RSS with tumor grade 1 and 4, RSS with tumor stage 1 and 4, as well as RSS with tumor small and large size.

**
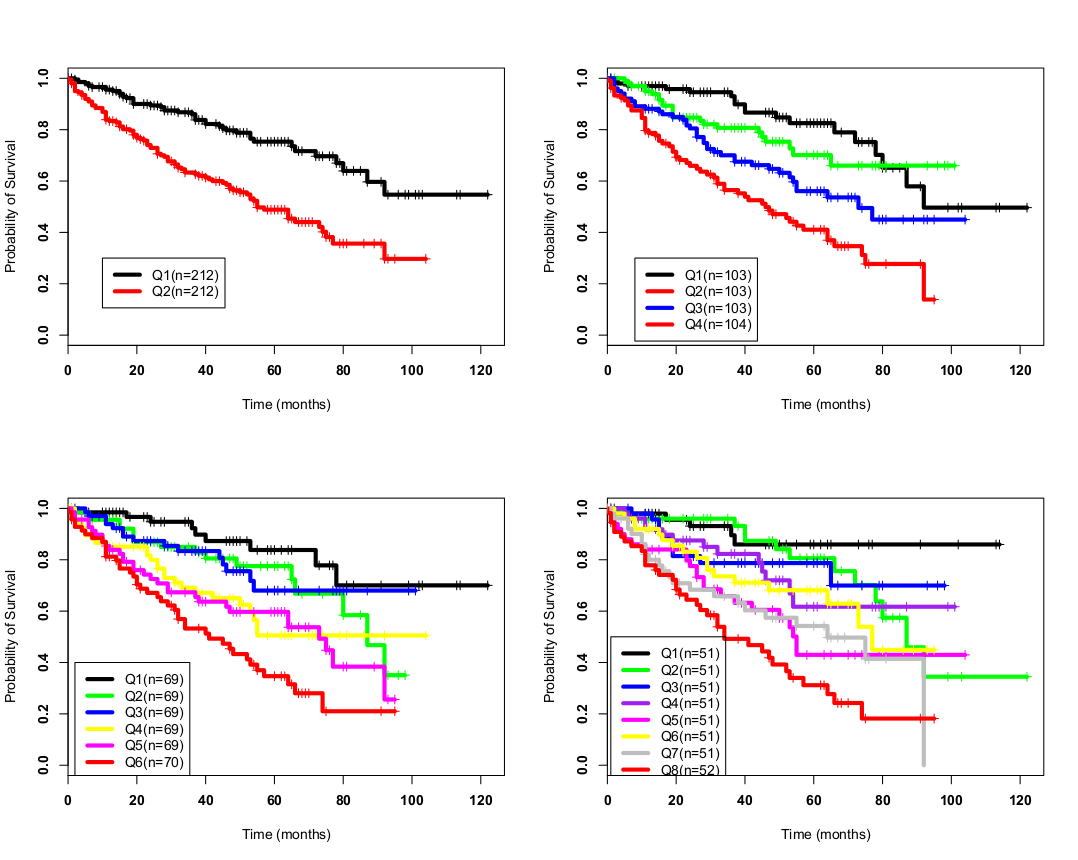
**

P=5.4e-09

P=1.53e-07

P=3.53e-06

P=7.53e-04

Supplementary Figure S5: Kaplan-Meier curves of cancer-specific survival for 414 TCGA samples. The samples are divided into 2, 4, 6 and 8 groups.


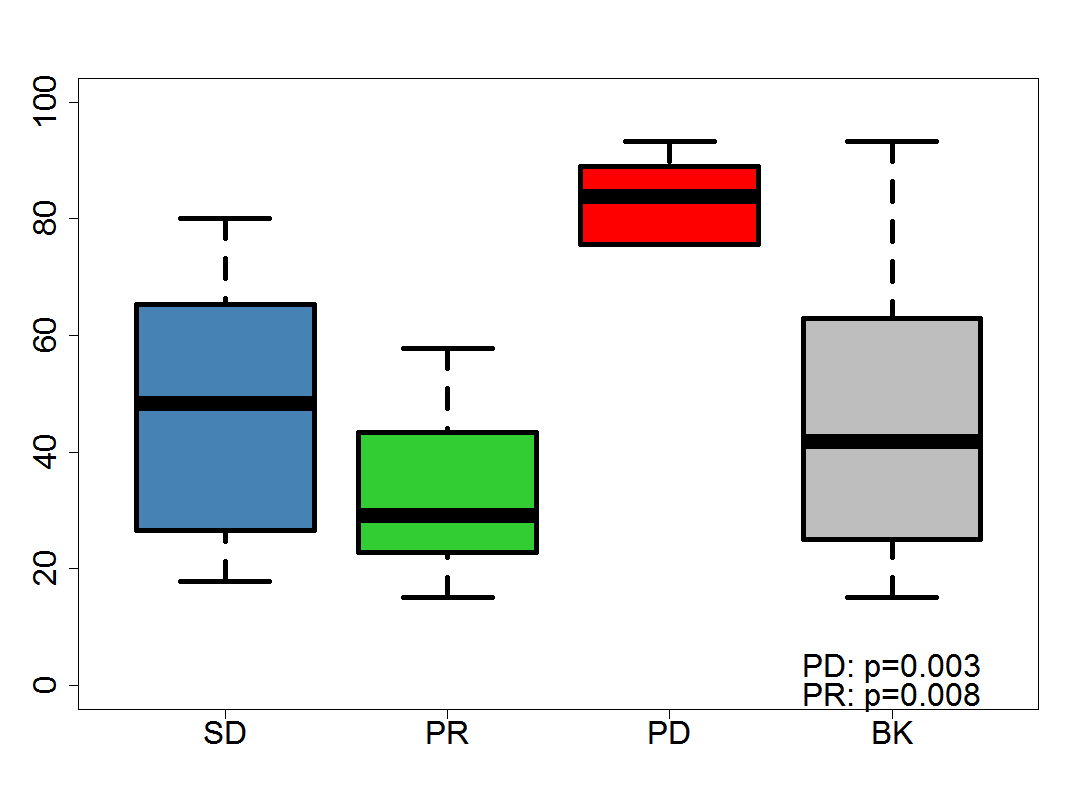


**Clear Score**

PR vs. BK (p=0.08)

SD vs. BK (p=0.034)

PD vs. BK (p=0.003)

PR vs. PD (p=0.000149)

Supplementary Figure S6: Boxplot of CLEAR score distribution in the patients with stable disease (SD), complete or partial response (CR/PR) and progressive disease (PD) with dataset of E-MTAB-3267. Mann–Whitney U test was used to compare the significance of CLEAR score with patients of SD, PR, PD and BK (all sample sets).

BK: all sample sets.


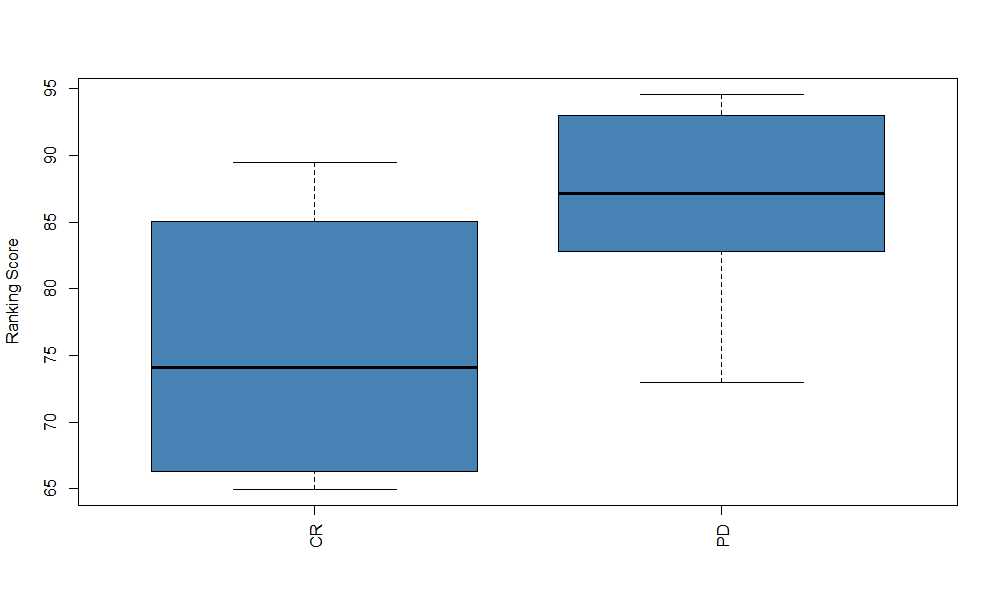


**Clear Score**

Supplementary Figure S7: Boxplot of CLEAR score distribution in the patients with complete response (CR) and progressive disease (PD) with IL-2 treatment in our dataset (Supplemental Table S4). Mann–Whitney U test was used to compare the significance of CLEAR score with patients of CR and PD (p = 0.05).


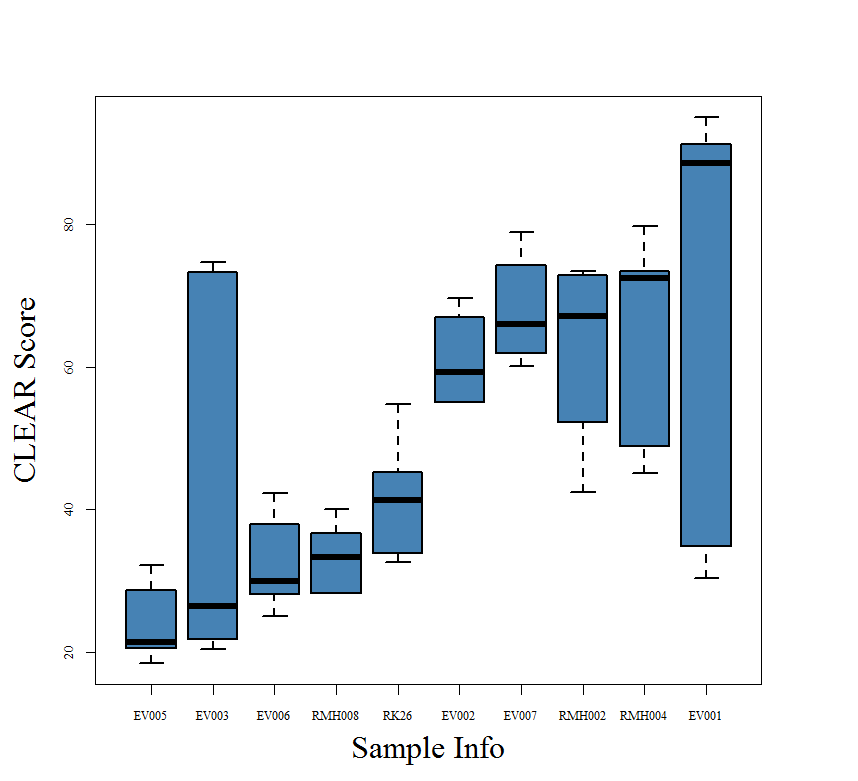


Supplementary Figure S8: Boxplot of CLEAR score distribution 65 regions from 10 ITH samples. The result showed when the median risk scores increased, the sub-samples tend to have more divergent CLEAR score.


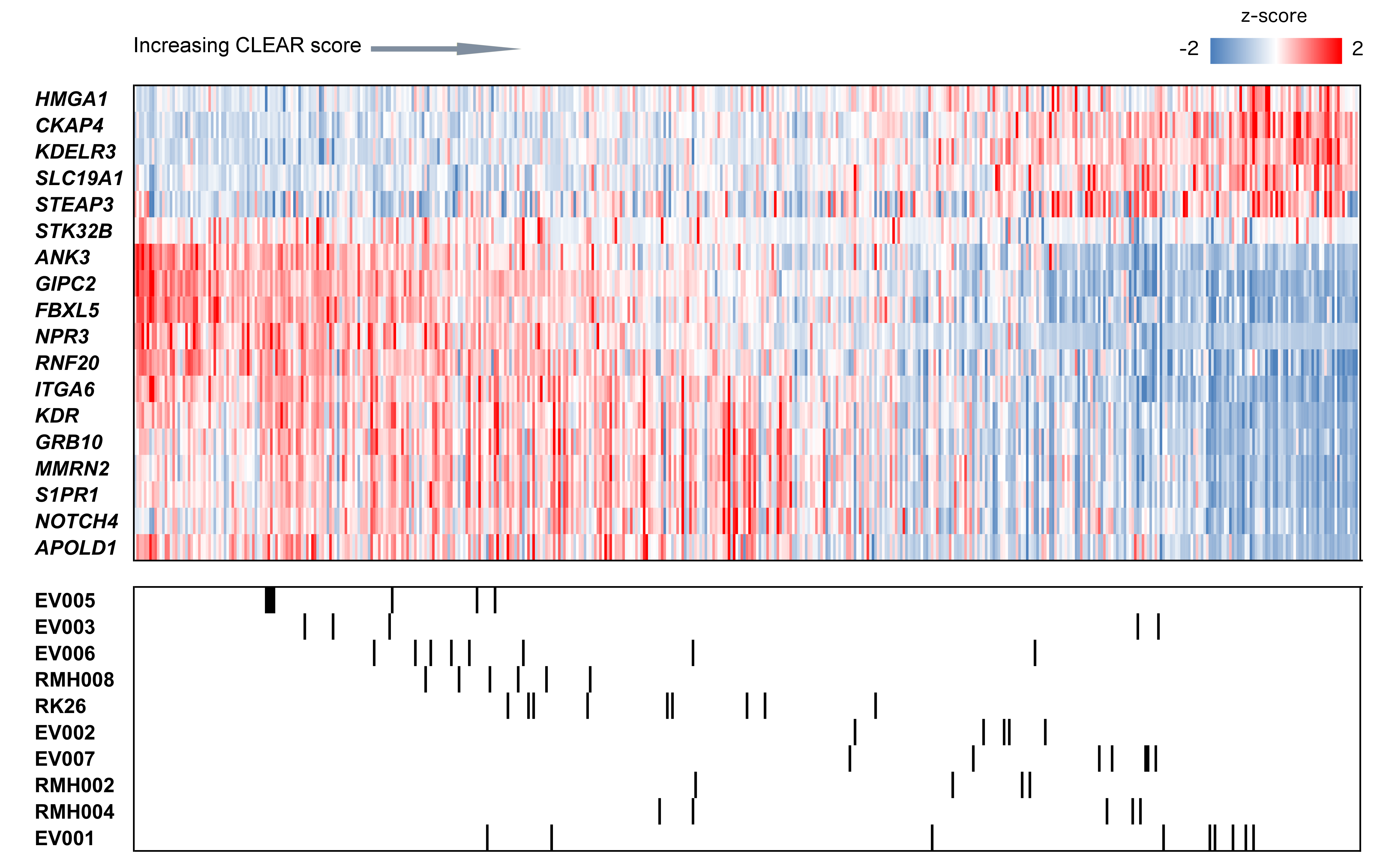


Supplementary Figure S9: Expression Heterogeneity and Clinical Outcomes in in ccRCC

A total of 479 gene expression profiles are presented here, ranked from left to right in ascending order by CLEAR score. This dataset comprises 414 expression profiles of individual TCGA tumors combined with 65 intratumoral regions sampled from 10 samples. For the lower panel, individual vertical black bars correspond to specific intratumoral region, which are matched to the 18-transcript expression in upper panel from the same corresponding region. From the lower panel, it may be seen that most, but not all, tumors have regions with relatively similar CLEAR scores. Median absolute deviation (MAD) results are found in Supplementary Table S5.


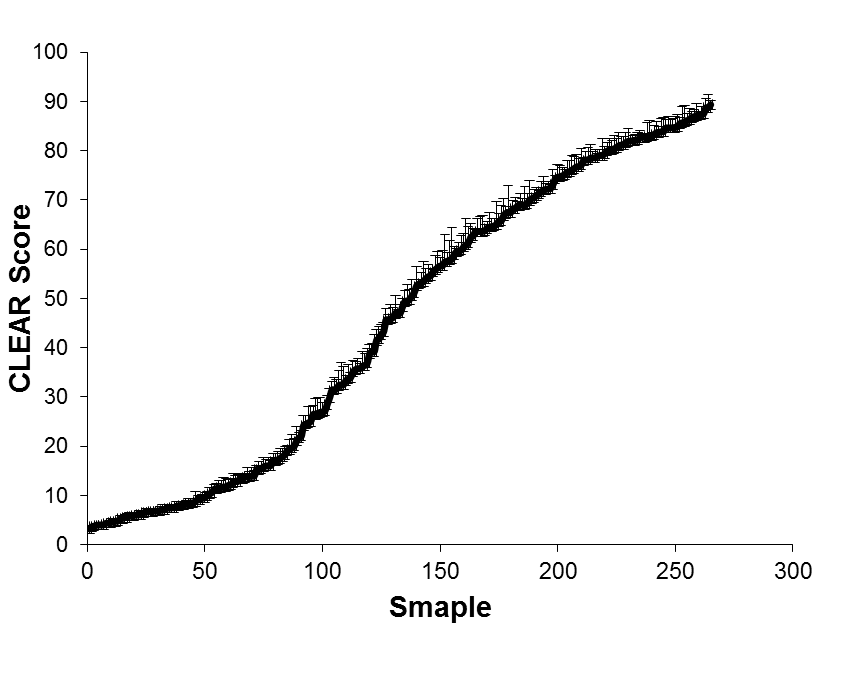


Supplementary Figure S10: Variation of CLEAR score regarding to the different reference sample sets (RSSs). Represents the range of CLEAR scores based on 500 RSSs.


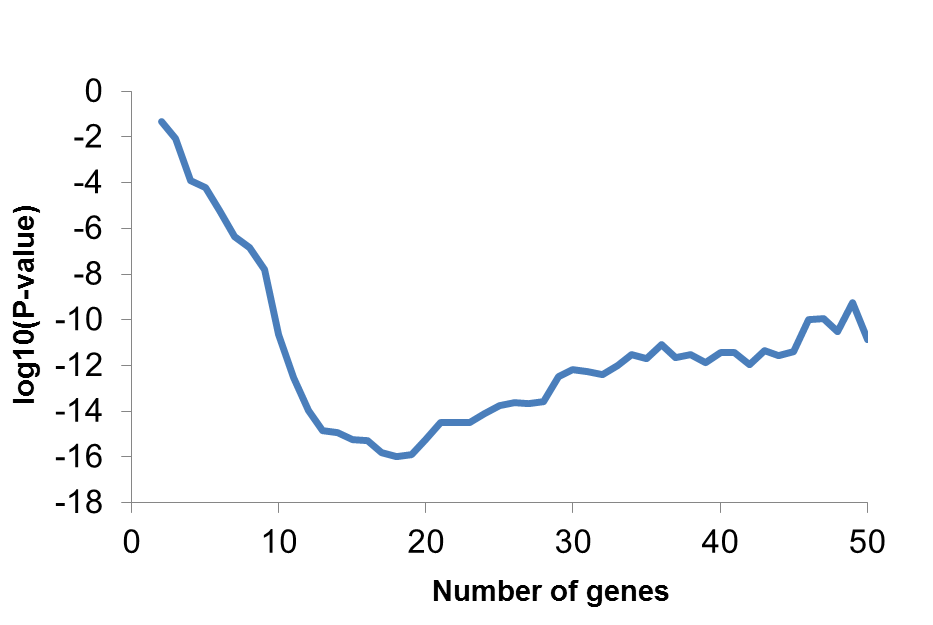


Supplementary Figure S11: Sensitivity analysis of Log-rank test with Cox proportional hazards model regarding to CLEAR Score. The result showed 18 genes has the best performance for prognostic estimates.

| Dataset | Platform | Samples |
| --- | --- | --- |
| GSE36895 | HG-U133_Plus_2 | 29 |
| GSE40435 | Illumina HumanHT-12 V4.0 | 104 |
| GSE25698 | Affymetrix Human Genome U133 Plus 2.0 | 32 |
| GSE20491 | Illumina HumanHT-12 V3.0 | 60 |
| GSE46699 | HG-U133_Plus_2 | 67 |
| GSE33093 | Agilent-014850 | 44 |
| GSE53757 | HG-U133_Plus_2 | 73 |
| GSE14994 | HT_HG-U133A | 70 |
| GSE16449 | Agilent-014850 | 51 |
| E-MTAB-3267 | Affymetrix GeneChip Human Gene  1.0 ST Array | 53 |
| GSE22541 | HG-U133_Plus_2 | 68 |
| TCGA | RNA-seq | 414 |

Supplementary Table S1: ccRCC datasets collected from GEO database, EMBL-EBI and TCGA.

| GSE46669 | | | | | |
| --- | --- | --- | --- | --- | --- |
| Repeat times | 10 | 20 | 40 | 80 | 100 |
| No. of clusters | 3 | 3 | 3 | 3 | 3 |
| Pitem | 10% | 20% | 40% | 80% | 100% |
| No. of clusters | 2 | 2 | 3 | 3 | 3 |
| Pfeature | 10% | 20% | 40% | 80% | 100% |
| No. of clusters | 3 | 3 | 3 | 3 | 3 |
| Distance | Pearson | Spearman | Euclidean |  |  |
| No. of clusters | 3 | 3 | 4 |  |  |
| GSE22541 | | | | | |
| Repeat times | 10 | 20 | 40 | 80 | 100 |
| No. of clusters | 4 | 4 | 4 | 4 | 4 |
| Pitem | 10% | 20% | 40% | 80% | 100% |
| No. of clusters | 4 | 4 | 4 | 4 | 4 |
| Pfeature | 10% | 20% | 40% | 80% | 100% |
| No. of clusters | 2 | 2 | 4 | 4 | 4 |
| Distance | Pearson | Spearman | Euclidean |  |  |
| No. of clusters | 4 | 3 | 4 |  |  |
| GSE14994 | | | | | |
| Repeat times | 10 | 20 | 40 | 80 | 100 |
| No. of clusters | 3 | 3 | 3 | 3 | 3 |
| Pitem | 10% | 20% | 40% | 80% | 100% |
| No. of clusters | 2 | 2 | 3 | 3 | 3 |
| Pfeature | 10% | 20% | 40% | 80% | 100% |
| No. of clusters | 3 | 3 | 3 | 3 | 3 |
| Distance | Pearson | Spearman | Euclidean |  |  |
| No. of clusters | 3 | 3 | 3 |  |  |
| GSE36895 | | | | | |
| Repeat times | 10 | 20 | 40 | 80 | 100 |
| No. of clusters | 2 | 2 | 2 | 2 | 2 |
| Pitem | 10% | 20% | 40% | 80% | 100% |
| No. of clusters | 2 | 2 | 3 | 3 | 3 |
| Pfeature | 10% | 20% | 40% | 80% | 100% |
| No. of clusters | 2 | 2 | 2 | 2 | 2 |
| Distance | Pearson | Spearman | Euclidean |  |  |
| No. of clusters  (Continue) | 2 | 2 | 2 |  |  |
| GSE40435 | | | | | |
| Repeat times | 10 | 20 | 40 | 80 | 100 |
| No. of clusters | 2 | 2 | 2 | 2 | 2 |
| Pitem | 10% | 20% | 40% | 80% | 100% |
| No. of clusters | 2 | 2 | 3 | 3 | 3 |
| Pfeature | 10% | 20% | 40% | 80% | 100% |
| No. of clusters | 3 | 3 | 3 | 3 | 3 |
| Distance | Pearson | Spearman | Euclidean |  |  |
| No. of clusters | 2 | 3 | 2 |  |  |
| GSE16449 | | | | | |
| Repeat times | 10 | 20 | 40 | 80 | 100 |
| No. of clusters | 3 | 3 | 3 | 3 | 3 |
| Pitem | 10% | 20% | 40% | 80% | 100% |
| No. of clusters | 2 | 2 | 3 | 3 | 3 |
| Pfeature | 10% | 20% | 40% | 80% | 100% |
| No. of clusters | 3 | 3 | 3 | 3 | 3 |
| Distance | Pearson | Spearman | Euclidean |  |  |
| No. of clusters | 3 | 3 | 3 |  |  |
| GSE53757 | | | | | |
| Repeat times | 10 | 20 | 40 | 80 | 100 |
| No. of clusters | 2 | 2 | 2 | 2 | 2 |
| Pitem | 10% | 20% | 40% | 80% | 100% |
| No. of clusters | 2 | 2 | 3 | 3 | 3 |
| Pfeature | 10% | 20% | 40% | 80% | 100% |
| No. of clusters | 2 | 2 | 2 | 2 | 2 |
| Distance | Pearson | Spearman | Euclidean |  |  |
| No. of clusters | 2 | 2 | 3 |  |  |
| TCGA | | | | | |
| Repeat times | 10 | 20 | 40 | 80 | 100 |
| No. of clusters | 3 | 3 | 3 | 3 | 3 |
| Pitem | 10% | 20% | 40% | 80% | 100% |
| No. of clusters | 3 | 3 | 3 | 3 | 3 |
| Pfeature | 10% | 20% | 40% | 80% | 100% |
| No. of clusters | 3 | 3 | 3 | 3 | 3 |
| Distance | Pearson | Spearman | Euclidean |  |  |
| No. of clusters  (Continue) | 3 | 3 | 3 |  |  |
| E-MTAB-3267 | | | | | |
| Repeat times | 10 | 20 | 40 | 80 | 100 |
| No. of clusters | 3 | 3 | 3 | 3 | 3 |
| Pitem | 10% | 20% | 40% | 80% | 100% |
| No. of clusters | 3 | 3 | 3 | 3 | 3 |
| Pfeature | 10% | 20% | 40% | 80% | 100% |
| No. of clusters | 3 | 3 | 3 | 3 | 3 |
| Distance | Pearson | Spearman | Euclidean |  |  |
| No. of clusters | 3 | 3 | 3 |  |  |

Supplementary Table S2: Summary of number of clusters of ccRCC using consensus clustering sensitivity analysis by varying the key parameters including pfeature (from 10% to 100%), pitem (from 10% to 100%), iteration (from 10 to 100), distance (pearson correlation, spearmen and euclidean distance) and linkage (ward, complete and average). The number of clusters are determined by observation of the cumulative distribution function (CDF) and the Delta_A(K) curve.

| Sample | Score | Sarcomatoid status |
| --- | --- | --- |
| Sample_1 | 30.67 | Renal Cell Carcinoma (Sarcomatoid) |
| Sample_29 | 42.39 | Renal Cell Carcinoma (Sarcomatoid) |
| Sample_16 | 86.37 | Renal Cell Carcinoma (Sarcomatoid) |
| Sample_172 | 95.85 | Renal Cell Carcinoma (Sarcomatoid) |
| Sample_72 | 89.71 | Renal Cell Carcinoma (Sarcomatoid) |
| Sample_45 | 88.44 | Renal Cell Carcinoma (Sarcomatoid) |
| Sample_118 | 97.73 | Renal Cell Carcinoma (Sarcomatoid) |
| Sample_169 | 88.47 | Renal Cell Carcinoma (Sarcomatoid) |
| Sample_141 | 91.17 | Renal Cell Carcinoma (Sarcomatoid) |
| Sample_44 | 93.23 | Renal Cell Carcinoma (Sarcomatoid) |
| Sample_2 | 96.62 | Renal Cell Carcinoma (Sarcomatoid) |
| Sample_77 | 95.13 | Renal Cell Carcinoma (Sarcomatoid) |
| Sample_42 | 85.85 | Renal Cell Carcinoma (Sarcomatoid) |
| Sample_151 | 95.84 | Renal Cell Carcinoma (Sarcomatoid) |

Supplementary Table S3: CLEAR score of Sarcomatoid Renal Cell Carcinoma samples.

| Sample | Normalization-score | IL-2 response |
| --- | --- | --- |
| Sample_239 | 80.68 | CR |
| Sample_241 | 64.97 | CR |
| Sample_242 | 67.57 | CR |
| Sample_240 | 89.47 | CR |
| Sample_245 | 72.98 | PD |
| Sample_254 | 82.77 | PD |
| Sample_244 | 83.65 | PD |
| Sample_256 | 90.68 | PD |
| Sample_247 | 94.58 | PD |
| Sample_255 | 93.01 | PD |

Supplementary Table S4: CLEAR score of ccRCC patients with IL-2 treatment in our internal datasets.

| Patient | MAD | Non- synonymous mutation | Driver SCNA  Heterogeneity (%) |
| --- | --- | --- | --- |
| EV005 | 4.38 | 54.2 | 66.7 |
| EV003 | 8.97 | 41.1 | 60 |
| EV006 | 4.29 | 28 | 33.3 |
| RMH008 | 6.15 | 67.9 | 50 |
| RK26 | 7.11 | 92 | 60 |
| EV002 | 6.29 | 70.9 | 77.8 |
| EV007 | 6.80 | 65.2 | 87.5 |
| RMH002 | 8.48 | 72.2 | 87.5 |
| RMH004 | 8.79 | 87.1 | 83.3 |
| EV001 | 9.04 | 68 | 92.3 |
| All the patients | 25.60 | NA | NA |

Supplementary Table S5: Median absolute deviation (MAD) of CLEAR score in each patient was used as an indicator for measurement of the variation of intratumoral heterogeneity (totally ten patients). Percentage of heterogeneous nonsynonymous mutation, percentage of heterogeneous driver copy number (CNA) aberrations are from the paper” Intratumor heterogeneity and branched evolution revealed by multiregion sequencing”.

| Number of samples |  | 265 |
| --- | --- | --- |
| Age | Range | 18-84 |
|  | Median | 63 |
| Gender- n (%) | Male | 101(38.1%) |
|  | Female | 161 (60.8%) |
| Stage – n (%) | I | 41 (15.4%) |
|  | II | 12 (4.5%) |
|  | III | 28 (10.6%) |
|  | IV | 44 (16.6%) |
| Tumor M stage – n (%) | 0 | 57 (21.5%) |
|  | 1 | 44(16.6%) |
|  | X | 113 (42.6%) |
| Tumor Grade – n (%) | 1 | 22 (8.3%) |
|  | 2 | 90 (34.0%) |
|  | 3 | 95 (35.8%) |
|  | 4 | 49 (18.5%) |
| Primary tumor size | Range | 1.4-29 |
|  | Median | 6.5 |
|  |  |  |
|  |  |  |

Supplementary Table S6: Clinical information of 265 datasets.

**References**

1. Brannon AR, Reddy A, Seiler M, et al: Molecular Stratification of Clear Cell Renal Cell Carcinoma by Consensus Clustering Reveals Distinct Subtypes and Survival Patterns. Genes Cancer 1:152-163, 2010

2. Brooks SA, Brannon AR, Parker JS, et al: ClearCode34: A prognostic risk predictor for localized clear cell renal cell carcinoma. Eur Urol 66:77-84, 2014

3. Beuselinck B, Job S, Becht E, et al: Molecular subtypes of clear cell renal cell carcinoma are associated with sunitinib response in the metastatic setting. Clin Cancer Res 21:1329-39, 2015

4. Tan MH, Kanesvaran R, Li H, et al: Comparison of the UCLA Integrated Staging System and the Leibovich score in survival prediction for patients with nonmetastatic clear cell renal cell carcinoma. Urology 75:1365-1370; 1370 e1-3, 2010

5. Harrell FE, Jr., Lee KL, Mark DB: Multivariable prognostic models: issues in developing models, evaluating assumptions and adequacy, and measuring and reducing errors. Stat Med 15:361-87, 1996
